# Supplementary material for: Chromothripsis during telomere crisis is independent of NHEJ, and consistent with a replicative origin
Source: Genome Res. 2019 May;29(5):737–49. doi: 10.1101/gr.240705.118 (PMC6499312; doi:10.1101/gr.240705.118)
Supplement: Supplemental Material [file supp_gr.240705.118_Supplemental_file_1.zip › contigs/annotated_contigs/DB113/contig.2.DB113_length_743_mean_cov_12.8398384926.docx]

**DB113_length_743_mean_cov_12.8398384926**

```
CGTCTAAAAACAAAAAACAAAGAACAAACACGAAAAAACTTAGGCCAGGTGCAATGGCTCGCGCCTGTAATCATAGCACTTTGGGAGGC
 >chr1:21898158-21898578 + E=7e-238 p=9e-03
CAAGGCAGGTTGATCACTTGAGCCCAGGAGTTTGAGACCAGCCTGGGAAACATAGCGAGACCTCAATTCTACAAAAATTTTTTAAAAAC

TGGCCAGGTGTGGTGGCGCACACCTTTAGTCCCAGCTACTCAGGAGGCTGAGGTAGGAGGATCCACCTGAGCCCAGGAGGTTGAGGCTG

CAGTGAGTTGTGATTATGCCACTGCACTCCAGCCTGGGTGACAGAGCAAGACCCTATCTTAAAAAAAAGAAAGAAAGAAAACATTAGCC

AGGTGTGGTGGTGCCAGCCACTCAGGAAGCTGACGTGGGAGGCTTGCTTGAGCCCAAGAGT|TTG|GAAGGACACCAGAATCAGAAGCA
                                                              >chr1:21899206-21899532 +
CCAGCATGGGACACATGGAACTTTGTGCCATGGGCAAGGAATGGATCATTCTCCTTATGTCACAAGTGAGAAAACGGAGCCCAGTGAGC
 E=3e-181
AGGAGGGACAATGATTCCAAACCTTCTGCTGTTTCGCTGCACAGGCCACCGTGCCCTTCAAATCCAACATGTAAATGCACACACACTCT

AGCGGCCCCATCGAGTCCAGGCTGTGACCCTCTGCCTTGGGGCCACAGTGCTGTGTGCTGAGCCCTGCAGGCTGGAGGCAAACTGACTG

ATGGATTTGAACCCCAGATCTGCCACTTCTCAG
```
